# Does Gender of Administrator Matter? National Study Explores U.S. University Administrators' Attitudes About Retaining Women Professors in STEM

**DOI:** 10.3389/fpsyg.2017.00700

**Published:** 2017-05-22

**Authors:** Wendy M. Williams, Agrima Mahajan, Felix Thoemmes, Susan M. Barnett, Francoise Vermeylen, Brian M. Cash, Stephen J. Ceci

**Affiliations:** Department of Human Development, Cornell University Ithaca, NY, United States

**Keywords:** underrepresentation of women, women in science, administrator gender, retention strategies, work-life balance, gender bias

## Abstract

Omnipresent calls for more women in university administration presume women will prioritize using resources and power to increase female representation, especially in STEM fields where women are most underrepresented. However, empirical evidence is lacking for systematic differences in female vs. male administrators' attitudes. Do female administrators agree on which strategies are best, and do men see things differently? We explored United States college and university administrators' opinions regarding strategies, policies, and structural changes in their organizations designed to increase women professors' representation and retention in STEM fields. A comprehensive review of past research yielded a database of potentially-effective, recommended policies. A survey based on these policies was sent to provosts, deans, associate deans, and department chairs of STEM fields at 96 public and private research universities across the U.S. These administrators were asked to rate the quality and feasibility of each strategy; 474 provided data, of which 334 contained complete numerical data used in the analyses. Our data revealed that female (vs. male) administrators believed the 44 strategies were higher in *quality* overall—but not higher in *feasibility*—with 9 strategies perceived differently by women and men, after imposing conservative statistical controls. There was broad general agreement on the *relative-quality* rankings of the 44 strategies. Women (vs. men) gave higher quality ratings to increasing the value of teaching, service, and administrative experience in tenure/promotion decisions, increasing flexibility of federal-grant funding to accommodate mothers, conducting gender-equity research, and supporting shared tenure lines enabling work-life balance. Women (vs. men) believed it was more feasible for men to stop the tenure clock for 1 year for childrearing and for universities to support requests for shared tenure lines, but less feasible for women to chair search committees. Our national survey thus supported the belief that placing women into administration creates greater endorsement of strategies to attract and retain women in STEM, although the effectiveness of these strategies was outside the scope of this research. Topics of disagreement between women and men are potentially important focuses of future policy, because female administrators may have insights into how to retain women that male administrators do not share.

## Introduction

**Figure 1:**
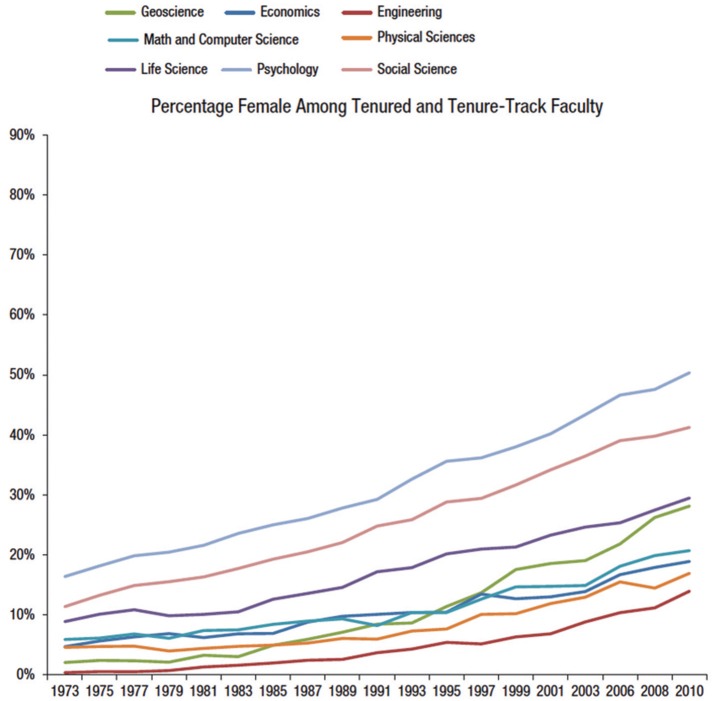
**Percentage female among tenure-track and tenured faculty from 1973 to 2010 as a function of field**. Values shown are weighted percentages. Data drawn from the National Science Foundation's Survey of Doctorate Recipients (adapted from Ceci et al., 2014).

Much has been written about the status of women in academic science (e.g., Ginther and Kahn, 2009, 2015; Ceci and Williams, 2010a,b; Williams and Ceci, 2012, 2015; Ceci et al., 2017). To be sure, women have made substantial progress in several STEM fields over the past two decades (e.g., Xie and Shauman, 2003; Hill et al., 2010). For example, female assistant professors are now at or above parity in psychological science and in most social sciences, and they are approaching parity in biological sciences (Ceci et al., 2014). However, women remain less numerous at senior ranks in all fields, and in the mathematically-intensive fields—physics, chemistry, computer science, engineering, economics, and geosciences—women occupy fewer than 20% of combined tenured and tenure-track professorships, as can be seen in Figure 1. Women's underrepresentation in academic science has led to the publication of articles, chapters, and books focusing on women's specific, practical, day-to-day needs in their colleges and universities, in the hope of addressing these needs through specific policies and strategies designed to better accommodate women and families (e.g., Williams and Ceci, 2012; Williams et al., 2013, 2015; Ceci and Williams, 2015; Jones et al., 2016).

Once hired, women face formidable challenges in academic science, which underscore the need for ongoing strategies and policies to address women's daily needs as professors. One key issue concerns research productivity and how the academic work environment may hinder women's success (Raj et al., 2016). Women professors publish fewer articles, chapters, and books than their male counterparts, a situation that may have implications for sex differences in hiring, salary, and promotion. Numerous researchers have documented productivity differences, using a variety of measures. Women publish less than men, starting in graduate school, and extending through the postdoctoral and pre-tenure years (Ceci et al., 2009).

**Figure 2:**
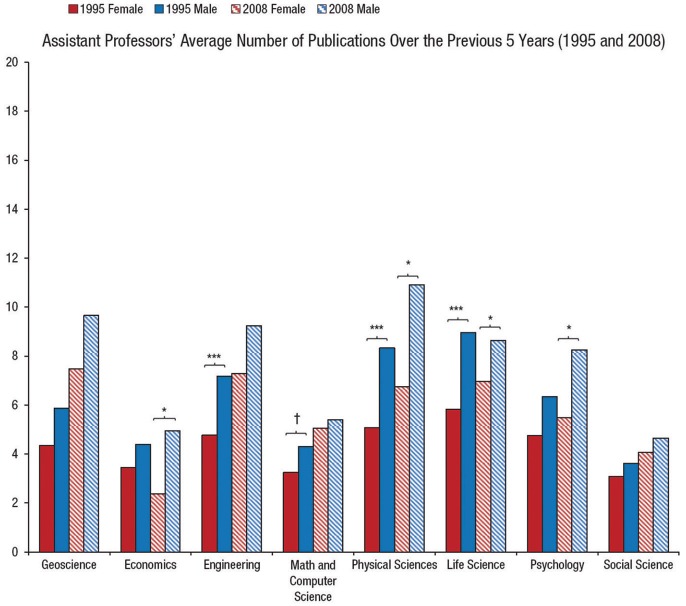
**Women vs. Men Assistant Professors: average number of publications over prior 5 years (adapted from Ceci et al., 2014)**. ^*^p < 0.05, †*p* < 0.01, ^***^p < 0.001.

Assistant professors represent the future of the academy; thus, it is interesting to examine trends in male and female assistant professors' productivity over the past 20 years. Elsewhere we have shown that in many fields, assistant professors of both sexes are publishing more articles in 2008 than in 1995, with some notable exceptions (Ceci et al., 2014). The average difference in publications by gender for assistant professors is 2.1 articles more for men than for women, which is equivalent to 27% of the total male assistant professor publications over the 5-year period. Figure 2 shows these differences by field. As can be seen, there is no clear-cut temporal trend; in some disciplines women's productivity increased between 1995 and 2008 while in others it declined, vis-a-vis men's productivity. On net, however, women published less in both periods. In each field in both 1995 and 2008, point estimates indicate that the average man published more than the average woman. The largest, statistically significant productivity gaps for assistant professors in 1995 were in engineering, life sciences, math/computer science, and physical science. By 2008, however, the fields of engineering and math/computer science saw these gaps close to the point at which they were no longer statistically significant. In life science by 2008, the gap narrowed but remained statistically significant, whereas in physical science the gap actually grew larger (for details please see pp. 103–107 of Ceci et al., 2014).

Data such as these have motivated administrators and gender-equity advocates to lobby for policies to aid women in the aftermath of childbirth or adoption, such as paid leaves, supplemental funding on grants to hire postdocs to run labs, and paid conference travel for childcare workers. However, it is not clear that the publishing gaps are causally related to family demands, because they exist among single, childless men and women as well (Williams and Ceci, 2012). Although the gap also appears among assistant professors at R1 institutions, with similar teaching responsibilities, it seems largely the result of sex differences in institutional resources, with women disproportionately more likely to work at small teaching-intensive institutions and men at research-intensive ones with greater resources for research (Ceci and Williams, 2011).

Unsurprisingly, women scientists in the academy are more likely to express dissatisfaction with aspects of their work that may be indirectly related to their underrepresentation and lower productivity. There are reports of an unwelcoming, “chilly” climate, indifferent attitudes toward family-work balance, and harassment, all of which may undermine women's success and persistence in the professoriate. Specifically, surveys of faculty indicate that the vast majority of women in science continue to describe an unwelcoming climate, including outright harassment (e.g., Ecklund and Lincoln, 2011). Coinciding with growth in their numbers, women scientists have reported being subjected to various barriers and challenges. Williams et al. (2014) reported the results of a survey in which they recruited 557 women scientists through the Association for Women in Science. Virtually all of the women claimed to have been victims of at least one of five biases they were asked about (e.g., sexual harassment; backlash for exhibiting stereotypically masculine behaviors such as assertiveness or expressing anger). Sixty-four percent of respondents with children reported a stigma when women took parental leave or stopped the tenure clock, leading the authors to conclude: “Motherhood appears to be a no-win proposition for many women in STEM” (Williams et al., 2014, p. 5). (Interestingly, motherhood worked both ways, with women without children also reporting dissatisfaction over being expected to work longer hours to make up for the schedules of colleagues who do have children.).

Against this broad backdrop of increasing numbers of women in the STEM professoriate, but persistent problems with productivity and allegations of workplace issues that undermine success, we wondered whether the gender of administrators makes a difference in the climate women face in STEM academic science. Are units, departments, colleges, and even universities headed by women likely to endorse more or different interventions and policies to combat the leakage of women STEM faculty? Note that this framing of the question differs from the more common framing, which asks whether women in units led by women are more satisfied. This is because satisfaction can be due to the mere presence of a same-sexed administrator and have nothing to do with any specific policies or procedures she or he advocated. We were interested in knowing whether female administrators endorsed a different constellation of strategies to attract and retain women faculty than were endorsed by their male counterparts. In a search of the literature we found nothing to directly answer this question, so we did the study ourselves.

Could it be that the lower number of women in some fields is associated with less aggressive leadership related to the recruitment and retention of women? Based on NSF's most recent Survey of Doctorate Recipients (SDR), there are small but statistically-significant sex differences when all types of institutions are combined: Women are less likely to be deans, directors, or department chairs (12.1 vs. 15.1%; *p* < 0.01); however, they are equally likely to be presidents, provosts, and chancellors (1.2 vs. 1.2%). Thus, the question suggests itself: Do departments, colleges, and universities that are headed by women endorse female-friendly practices that male administrators are less likely to endorse?

Some qualitative data suggest that female administrators provide a sense of social capital in the workplace for women that male administrators may not (Smith, 2014). For example, based on interviews, Dunn et al. (2014) reported widely-varying administrative styles of men and women administrators. Intensive interviews they conducted with 19 women in STEM fields at five universities revealed a sense of isolation related to a relative lack of social capital (e.g., connections, tacit knowledge, membership in networks, and possession of material resources). Successful women administrators' style of leadership (building social capital and combining both agentic assertiveness and communal warmth) may be better at communicating and breaking down such feelings of isolation (Eagly and Carli, 2007). Other evidence suggests that women and minorities respond best in more collaborative learning experiences, which is a distinctly female leadership style (see Gorman et al., 2010). Finally, Hough (2010) profiled the leadership styles of 183 female administrators at senior level positions, such as president, chancellor, vice president, and dean, at accredited institutions of higher education. She reported that effective administrators strive to increase a sense of community and collegiality.

In sum, social capital theory explains why having female administrators might work positively to attract and retain women in STEM; however, no direct data exist regarding whether female and male administrators actually endorse different strategies to attract and retain women in STEM. In the survey that follows we asked a national sample of administrators to rate various female-friendly strategies that have been proposed in the literature. Do women and men differ in their support of these interventions? And, what can we learn regarding strategies that were supported by both genders, as opposed to strategies endorsed more by one gender than the other?

## Method

We began by compiling a list of potential strategies for attracting and retaining women in academic science. We gathered these strategies from articles in the PsycINFO database and from Google/Google Scholar searches, found via search terms comprised of various combinations of the words “women,” “science,” “STEM,” “underrepresentation,” “gender,” “professor,” “academic,” “hiring,” “tenure,” “retention,” “strategies,” “policies,” “procedures,” “family friendly,” and “university administrator,” (for example, “women in science,” “women in STEM,” “STEM retention,” “academic retention strategies,” and so on). We sifted through 206 articles (by which point we were encountering substantial redundancies in strategies mentioned and/or advocated). We also followed additional leads found in these articles' reference sections to point us to mentions of further potential strategies. Our overarching goal was to compile a lengthy, representative list of recommendations for increasing the presence and persistence of women in academic science.

Based on this corpus of research, we next whittled the list of policies and recommendations to remove redundancies. For example, numerous researchers recommended establishing committees to monitor women's progress (i.e., conducting institutional research on gender-equity issues; see, e.g., Committee on Maximizing the Potential of Women in Academic Science and Engineering, National Academy of Sciences, National Academy of Engineering, Institute of Medicine, 2007; National Research Council, 2009), and stopping the tenure clock for family formation (e.g., Goulden et al., 2009; Williams and Ceci, 2012). The resulting list of policies and recommendations was then sent to 24 natural and social science faculty across ranks, who were asked to comment on remaining redundancies and add any potential missing strategies. Our goal was to include the most important and often-mentioned strategies in a comprehensive list, which we then used as the basis for national empirical data collection. Based on the feedback from professors across ranks in six science and social science disciplines, the list of strategies was iteratively revised until we developed a final survey containing 44 strategies.

The final survey (see Table 1) was emailed to 1,529 administrators at 96 public and private research universities across the United States (see Table 2). The target population consisted of provosts, deans, associate deans, and department chairs of STEM fields at American Carnegie 1 research-oriented universities, formerly called R1s. These United States university administrators were asked for two responses to each policy—a rating of its *quality* and a rating of its *feasibility*. Ratings were based on a 9-point Likert scale, with 1 being the lowest score and 9 being the highest. Two-hundred-thirty of the individuals in our database had either left administration, retired permanently, gone on leave, changed universities or had otherwise been separated from their former positions. Our survey received 474 responses (36.5% response rate), of which 334 contained complete numerical data used in the analysis. The other 130 replies contained incomplete data, or responses that consisted of comments about the importance of retaining women, personal anecdotes, and so on, as opposed to complete sets of ratings (Note that we are not asserting that the sample was perfectly representative of the population of U.S. college and university administrators, only that the 334 administrators represented all 96 R1s). For each respondent, publicly-available data was gathered on her or his gender, title, and university type. Data were then de-identified to ensure anonymity of responses. Of the 334 respondents, 246 were men and 88 were women; there were 157 men and 34 women STEM department chairs, 38 men and 22 women associate deans, 42 men and 24 women deans, and nine men and eight women provosts, all from Carnegie 1 research universities.

**Table 1:** **STEM-administrator survey**.

Please rate each of the following policy ideas on a 1-to-9 scale for QUALITY and FEASIBILITY, in which 1 = extremely low, 3 = somewhat low, 5 = neutral, 7 = somewhat high, and 9 = extremely high. By QUALITY (“Q”) we mean: How good is this strategy, if the goal is to increase the number of women in traditionally-underrepresented STEM fields in the professoriate? By FEASIBILITY (“F”) we mean: How workable, cost-effective, and reasonable would this strategy be to implement?
**Addressing Gender Biases During Hiring**
Have a woman chair search committees whenever possible. Q___F___
Reward departments that hire women. Q___F___
Set gender goals for candidate pools. Q___F___
Set quotas for new lines: women-only lines until critical mass reached. Q___F___
Explore/endorse couples-hiring. Q___F___
Guarantee academic employment for professional spouses/partners. Q___F___
Instruct search committees to ignore family-related gaps in CVs. Q___F___
**Addressing Gender Biases After Hiring**
Set gender quotas (minimum thresholds) for promotion to higher levels of rank (e.g., full professor). Q___F___
Set gender quotas for important committees and administrative posts. Q___F___
For promotion, increase value of teaching and service plus administration. Q___F___
Conduct (and disseminate) institutional research on gender equity. Q___F___
**Attaining Tenure and Maintaining Productivity**
Provide fully-paid leave for giving birth (tenure-track women only): For 6 weeks? Q___F___ For 1 semester? Q___F___ For 1 year? Q___F___
Provide fully-paid leave for adoption/new parenthood (tenure-track women and men): For 6 weeks? Q___F___ For 1 semester? Q___F___ For 1 year? Q___F___
Provide teaching relief for new tenure-track parents: 1 semester? Q___F___ 1 year? Q___F___
Stop the tenure clock for raising children for up to 1 year per child: For mothers? Q___F___ For fathers? Q___F___
Change timing of tenure assessment to not coincide with peak fertility and childrearing demands. Q___F___
Allow option of changing from full-time to part-time tenure-track: Short Term (up to 1 year) Q___F___ Medium Term (2–5 years) Q___F___ Permanent Q___F___
Support requests for shared tenure lines (between partners). Q___F___
**Balancing Work and Family**
Provide on-campus childcare centers. Q___F___
Provide subsidies for on-campus or off-campus childcare services. Q___F___
Allow unpaid sabbaticals and leave of absences for both genders without penalty, for family-related reasons such as elder caretaking and issues with children. Q___F___
Offer family housing subsidies in regions where young families are priced out of the market. Q___F___
Use technology to allow women and men with children to work and attend meetings from home. Q___F___
Provide an academic role for women who have left professional positions to have children. Q__ F__
**Providing Leadership and Training Opportunities**
Provide equal opportunities for women and men to lead committees and research groups. Q___F___
Train department chairs on helping faculty manage work-life issues. Q___F___
Develop mentoring programs to reduce isolation of female faculty. Q___F___
Convene gender-equity workshops focusing on issues such as workplace climate and resource allocation. Q___F___
**Supporting Greater Flexibility for Federal Grants and Funding**
Support no-cost extensions for caregiving on grants and fellowships. Q___F___
Support part-time fellowships and grants. Q___F___
Support the deferred start of fellowships to allow for caregiving. Q___F___
Endorse supplements to offset PI's productivity loss due to family-related absences. Q___F___
Support conference and meeting grant supplements to cover cost of PI's dependent care travel (children's and childcare workers' expenses allowable). Q___F___
Support grants for retooling after maternity leave. Q___F___
Provide support to help faculty engaging in caregiving duties to catch up mid-career. Q___F___
Endorse supplemental funding for hiring postdocs to maintain momentum during family leaves. Q___F___

**Table 2:** **Universities in sample**.

Arizona State University, Brandeis University, Brown University, California Institute of Technology, Carnegie Mellon University, Case Western Reserve University, Colorado State University, Columbia University, Cornell University, Dartmouth University, Duke University, Emory University, Florida State University, Georgetown University, Georgia Institute of Technology, Harvard University, Indiana State University, Indiana University, Iowa State University, Johns Hopkins University, Kansas State University, Louisiana State University, Michigan State University, Massachusetts Institute of Technology, Montana State University, North Carolina State University, Northwestern University, New York University, Ohio State University, Oregon State University, Penn State University, Princeton University, Purdue University, Rensselaer Polytechnic Institute, Rice University, Rutgers University, Stanford University, SUNY Albany, SUNY Buffalo, SUNY Stony Brook, Texas A and M University, Tulane University, UC Berkeley, UC Davis, UC Denver, UC Irvine, UC Riverside, UC San Diego, UC Santa Barbara, UC Santa Cruz, UC Los Angeles, University of Alabama at Birmingham, University of Arizona, University of Cincinnati, University of Colorado at Boulder, University of Connecticut, University of Delaware, University of Florida, University of Georgia, University of Hawaii, University of Illinois at Chicago, University of Illinois at Urbana-Champaign, University of Iowa, University of Kansas, University of Kentucky, University of Maryland, University of Massachusetts—Amherst, University of Miami, University of Michigan, University of Minnesota, University of Missouri, University of Nebraska-Lincoln, University of New Mexico, University of North Carolina, University of Notre Dame, University of Pennsylvania, University of Pittsburgh, University of Rochester, University of South Carolina, University of South Florida, University of Southern California, University of Tennessee, University of Texas, University of Utah, University of Virginia, University of Washington, University of Wisconsin-Madison, Vanderbilt University, Virginia Polytechnic Institute, Washington State University, Washington University in St Louis, Wayne State University, Yale University, Yeshiva University.

## Results and discussion

**Figure A1:**
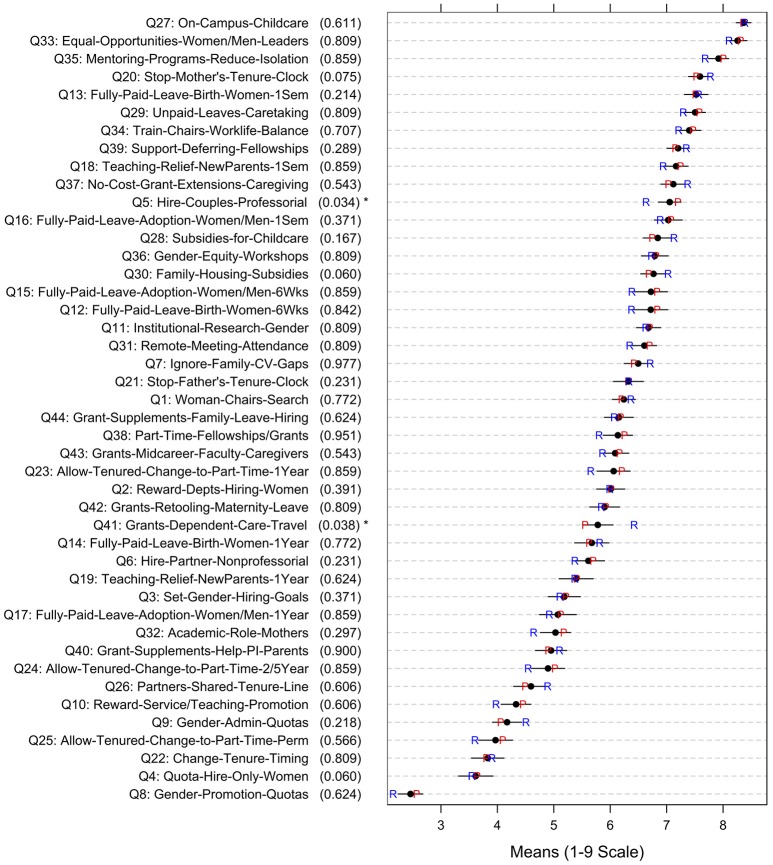
Strategy Quality Ratings: Overall Means, Confidence Intervals, and Means by University Type (P = Public; R = Private; adjusted ***p***-values in parentheses show significance level of comparison of item ratings by university type; ^*^p ≤ 0.05).

**Figure A2:**
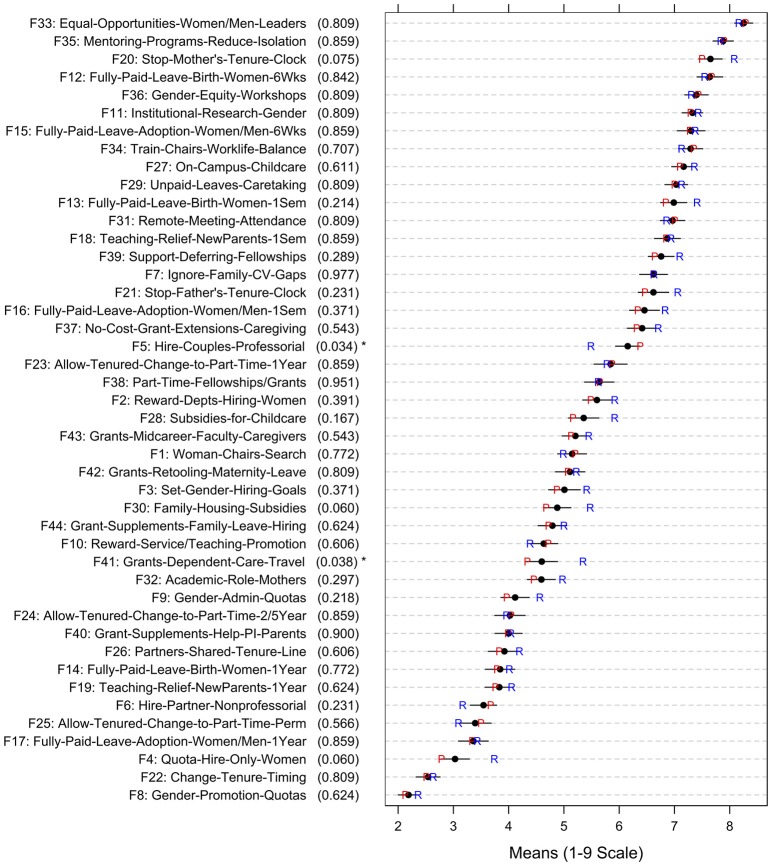
Strategy Feasibility Ratings: Overall Means, Confidence Intervals, and Means by University Type (P = Public; R = Private; adjusted ***p***-values in parentheses show significance level of comparison of item ratings by university type; ^*^p ≤ 0.05).

The analyses ranked strategies for their quality and feasibility, and examined whether administrator gender affected ratings of the policies. We also evaluated the impact of university type (public or private) and geographical location of institution on ratings of policies; location was not systematically related to strategy ratings, and results for university type, public vs. private, appear in Appendix (Figures A1, A2). For each set of comparisons (e.g., comparing all strategies across gender) we adjusted *p*-values using the conservative Benjamini and Hochberg (1995) false-discovery rate at a 5% level.

### Overall effect of strategy quality

A repeated-measures ANOVA was conducted on the quality ratings of the 44 strategies by the 334 respondents, to evaluate whether the mean ratings of items differed significantly across items. The result was highly significant—*F*_(16.02, 4236.45)_ = 81.65, *p* < 0.001, with Greenhouse-Geiser correction for violations of sphericity. This finding showed that respondents of both genders perceived strategies as varying systematically in quality, with broad general agreement concerning the strategies' relative rankings. Strategies discussed in the next section (“General Agreement about Relative Quality of Strategies”) are ones about which administrators of both genders agreed. Strategies characterized by sex differences in opinions are discussed in the subsequent section (“Gender Differences in Ratings of Strategy Quality or Feasibility”). Ratings of strategy quality correlated 0.98 with strategy feasibility, so we focus on quality ratings in this discussion of results, except for those few occasions when ratings of quality and feasibility differed, such as in the situation discussed below under “Gender Differences in Ratings of Strategy Quality or Feasibility.”

### General agreement about relative quality of strategies

The 44 strategies ranked by quality ratings are shown in Table 3, with the mean rating for each policy on a 1-to-9 scale (1 = extremely low, 3 = somewhat low, 5 = neutral, 7 = somewhat high, and 9 = extremely high). Twelve strategies had high mean quality ratings of 7.0 or more; we describe these strategies here, proceeding with a brief description of the balance of the strategies in order from highest to lowest quality.

**Table 3:** **Strategies for increasing/retaining women in STEM professoriate, listed from highest to lowest quality (*n* = 334 faculty respondents; 44 strategies rated on 1-to-9 scale in which 1 = extremely low, 3 = somewhat low, 5 = neutral, 7 = somewhat high, and 9 = extremely high)**.

1	Provide on-campus childcare centers. (Q27, *M* = 8.36)
2	Provide equal opportunity for women and men to lead committees and research groups. (Q33, *M* = 8.26)
3	Develop mentoring programs to reduce isolation of female faculty. (Q35, *M* = 7.92)
4	Stop tenure clock for raising children for up to 1 year per child. (Q20, *M* = 7.59)
5	Provide fully-paid leave for giving birth (tenure-track women only): For 1 semester. (Q13, *M* = 7.52)
6	Allow unpaid sabbaticals and leave of absences for both genders without penalty, for family-related reasons such as elder caretaking and issues with children. (Q29, *M* = 7.50)
7	Train department chairs on helping faculty manage work-life issues. (Q34, *M* = 7.40)
8	Support the deferred start of fellowships to allow for caregiving. (Q39, *M* = 7.20)
9	Provide teaching relief for new tenure-track parents: 1 semester. (Q18, *M* = 7.17)
10	Support no-cost extensions for caregiving on grants and fellowships. (Q37, *M* = 7.12)
11	Explore/endorse couples-hiring. (Q5, *M* = 7.05)
12	Provide fully-paid leave for adoption/new parenthood (tenure-track women and men): For 1 semester. (Q15, *M* = 7.03)
13	Provide subsidies for on-campus or off-campus childcare services. (Q28, *M* = 6.84)
14	Convene gender-equity workshops focusing on issues such as workplace climate and resource allocation. (Q36, *M* = 6.79)
15	Offer family housing subsidies in regions where young families are priced out of the market. (Q30, *M* = 6.77)
16	Provide fully-paid leave for adoption/new parenthood (tenure-track women and men): For 6 weeks. (Q15, *M* = 6.72)
17	Provide fully-paid leave for giving birth (tenure-track women only): For 6 weeks. (Q12, *M* = 6.72)
18	Conduct (and disseminate) institutional research on gender equity. (Q11, *M* = 6.68)
19	Use technology to allow women and men with children to work and attend meetings from home. (Q31, *M* = 6.61)
20	Instruct search committees to ignore family-related gaps in CVs. (Q7, *M* = 6.50)
21	Stop the tenure clock for raising children for up to 1 year per child: For fathers. (Q21, *M* = 6.32)
22	Have a woman chair search committees whenever possible. (Q1, *M* = 6.24)
23	Endorse supplemental funding for hiring postdocs to maintain momentum during family leaves. (Q44, *M* = 6.15)
24	Support part-time fellowships and grants. (Q38, *M* = 6.14)
25	Provide support to help faculty engaging in caregiving duties to catch up mid-career. (Q43, *M* = 6.09)
26	Allow option of changing from full-time to part-time tenure-track: Short Term (up to 1 year). (Q23, *M* = 6.06)
27	Reward departments that hire women. (Q2, *M* = 6.01)
28	Support grants for retooling after maternity leave. (Q42, *M* = 5.90)
29	Support conference and meeting grant supplements to cover cost of PI's dependent care travel (children's and childcare workers' expenses allowable). (Q41, *M* = 5.78)
30	Provide fully-paid leave for giving birth (tenure-track women only): For 1 year. (Q14, *M* = 5.67)
31	Guarantee academic employment for professional spouses/partners. (Q6, *M* = 5.61)
32	Provide teaching relief for new tenure-track parents: 1 year. (Q19, *M* = 5.40)
33	Set gender goals for candidate pools. (Q3, *M* = 5.19)
34	Provide fully-paid leave for adoption/new parenthood (tenure-track women and men): For 1 year. (Q17, *M* = 5.07)
35	Provide an academic role for women who have left professional positions to have children. (Q32, *M* = 5.03)
36	Endorse supplements to offset PI's productivity loss due to family-related absences. (Q40, *M* = 4.95)
37	Allow option of changing from full-time to part-time tenure-track: Medium Term (2–5 years). (Q24, *M* = 4.90)
38	Support requests for shared tenure lines (between partners). (Q26, *M* = 4.60)
39	For promotion, increase value of teaching and service plus administration. (Q10, *M* = 4.33)
40	Set gender quotas for important committees and administrative posts. (Q9, *M* = 4.17)
41	Allow option of changing from full-time to part-time tenure-track: Permanent. (Q25, *M* = 3.97)
42	Change timing of tenure assessment to not coincide with peak fertility and childrearing demands. (Q22, *M* = 3.83)
43	Set quotas for new lines: women-only lines until critical mass reached. (Q4, *M* = 3.62)
44	Set gender quotas (minimum thresholds) for promotion to higher levels of rank (e.g., full professor). (Q8, *M* = 2.46)

The highest-quality strategy was for universities to provide on-campus childcare centers (*M* = 8.36), which unsurprisingly is a priority for universities across the U.S., reflecting the challenges faced by women (and men) faculty with preschool-aged children. Offering equal opportunities for women and men to lead committees and research groups (*M* = 8.26) was also seen as an extremely high-quality strategy, as was developing mentoring programs to reduce isolation of female faculty (*M* = 7.92).

A policy that has become widely used over the past decade, stopping the tenure clock for raising children for up to 1 year per child (*M* = 7.59), was the next-highest-rated strategy. Providing fully-paid leave for giving birth for tenure-track women only, for a total of one semester, was seen as valuable (*M* = 7.52), as was allowing unpaid sabbaticals and leaves of absence for both genders without penalty, for family-related reasons such as elder caretaking and issues with children (*M* = 7.50).

In recognition of the role played by departmental-level administrators, respondents endorsed training for department chairs on helping faculty manage work-life issues (*M* = 7.40). Respondents also supported the deferred start of fellowships to allow for caregiving (*M* = 7.20), and providing of teaching relief for new tenure-track parents for one semester (*M* = 7.17). Another strategy related to caregiving was also endorsed: Supporting no-cost extensions for caregiving on grants and fellowships (*M* = 7.12). Institutions' need to explore and endorse couples-hiring to help resolve the two-body problem was also rated highly (*M* = 7.05). And finally, there was broad support for providing fully-paid leave for adoption and new parenthood, for tenure-track women and men, for one semester (*M* = 7.03).

Concomitant with the endorsement of providing on-campus childcare centers (discussed above), support was found for the importance of providing subsidies for childcare (*M* = 6.84), and family housing subsidies (*M* = 6.77). Both genders also believed that gender equity workshops were valuable (*M* = 6.79). On the topic of leaves for faculty becoming parents, respondents supported fully paid leave for adoption for women and men for 6 weeks (*M* = 6.72), as well as fully paid leave for giving birth for women, also for 6 weeks (*M* = 6.72). Also accommodating parents and those with travel and caretaking demands, we found support for the importance of allowing remote meeting attendance (*M* = 6.61). With children often comes a challenge to maintaining productivity, and we found support for the practice of ignoring family-related gaps in CVs (*M* = 6.50); that is, respondents agreed that someone with a total of 5 years on tenure track, one of which was spent on leave due to childcare, should be considered just 4 years on tenure track for purposes of setting the tenure clock. We also noted an endorsement of the policy of temporarily stopping the tenure clock for fathers (*M* = 6.32).

Note, again, that a rating of five signified neutral quality and a seven signified somewhat high quality. Having women chair searches was seen as a generally good quality strategy (*M* = 6.24), as was the awarding of part-time fellowships and grants to accommodate parents and academics with caregiving responsibilities (*M* = 6.14). Similarly, midcareer grants to faculty caregivers was rated above 6 (*M* = 6.09) as was the policy of allowing tenured faculty to go part-time for 1 year (*M* = 6.06). Rated just above 6 was the strategy of rewarding departments for hiring women (*M* = 6.01).

Between a neutral-quality rating of 5 and a slightly-high rating of 6, we found modest support for grants for retooling after maternity leave (*M* = 5.90), and funding fully paid leave for giving birth for women for 1 entire year (*M* = 5.67). Encouraging universities and colleges to hire faculty partners and spouses for non-professorial positions was also somewhat weakly endorsed (*M* = 5.61). Similarly, giving teaching relief to new parents for 1 year (*M* = 5.40) was weakly supported. Support was generally neutral for the practice of setting gender hiring goals (*M* = 5.19) and for offering fully paid leave for adoption to women and men faculty for 1 year (*M* = 5.07). Providing an academic role for mothers who used to be professors or who wish to participate in university life also was seen as a neutral strategy (*M* = 5.03).

Turning to a consideration of strategies deemed to be of relatively lower quality by the 334 respondents, six strategies had mean quality ratings below 5.0 (and no gender interactions affecting the interpretation of the results). We describe these strategies below, ordered from relatively better to relatively worse. Interestingly, one frequently-mentioned strategy widely acknowledged in the literature as being especially beneficial for women was seen by our respondents as being of relatively low quality: Allowing the option of changing from full-time to part-time tenure-track, over the medium term of 2–5 years (*M* = 4.90). It has been argued that women may wish to work part-time for a few years, during which they can have and raise children, then later segue back to full-time work when their children begin school. Yet our data call into question the wisdom of this life plan, at least from the administrators' point of view. Setting gender quotas for important committees and administrative posts (*M* = 4.17), and allowing the option of changing from full-time to part-time tenure-track on a permanent basis (*M* = 3.97), were also relatively weakly endorsed.

In another surprise, administrators did not support changing the timing of tenure assessment to avoid peak fertility and childrearing demands (*M* = 3.83). This strategy has been broadly advocated as an essential way to reduce pressure on women scholars, who are expected to amass a tenurable portfolio during the exact same years as when they tend to have children—in their thirties. The fact that women in science experience the confluence of the tenure clock and the biological clock, but that men in science simply do not share these limitations, is an inescapable aspect of the dilemma faced by female scholars. What, exactly, the academy can do to ameliorate this problem for women scholars remains a pressing question. Other weakly-endorsed strategies included setting gender quotas for new tenure lines and calling for women-only lines until a critical mass of women is reached (*M* = 3.62). Finally, the worst strategy of all was seen as setting gender quotas (minimum thresholds) for promotion to higher levels of rank (e.g., full professor *M* = 2.46).

### Gender differences in ratings of strategy quality or feasibility

**Figure 3:**
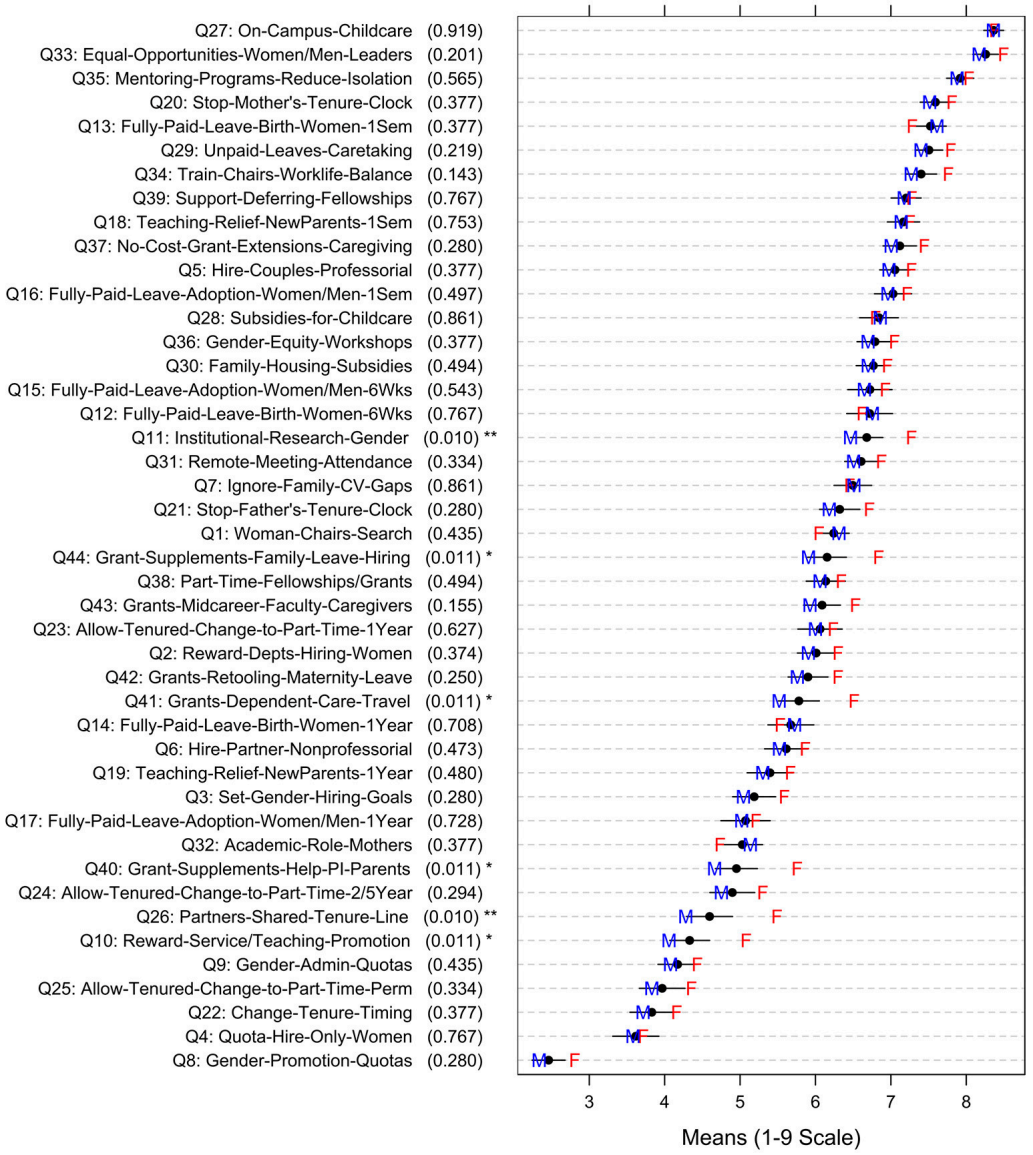
Strategy Quality Ratings: Overall Means, Confidence Intervals, and Means by Gender (F = Female; M = Male; adjusted *p*-values in parentheses show significance level of comparison of item ratings by gender; ^*^p ≤ 0.05, ^**^p ≤ 0.01).

**Figure 4:**
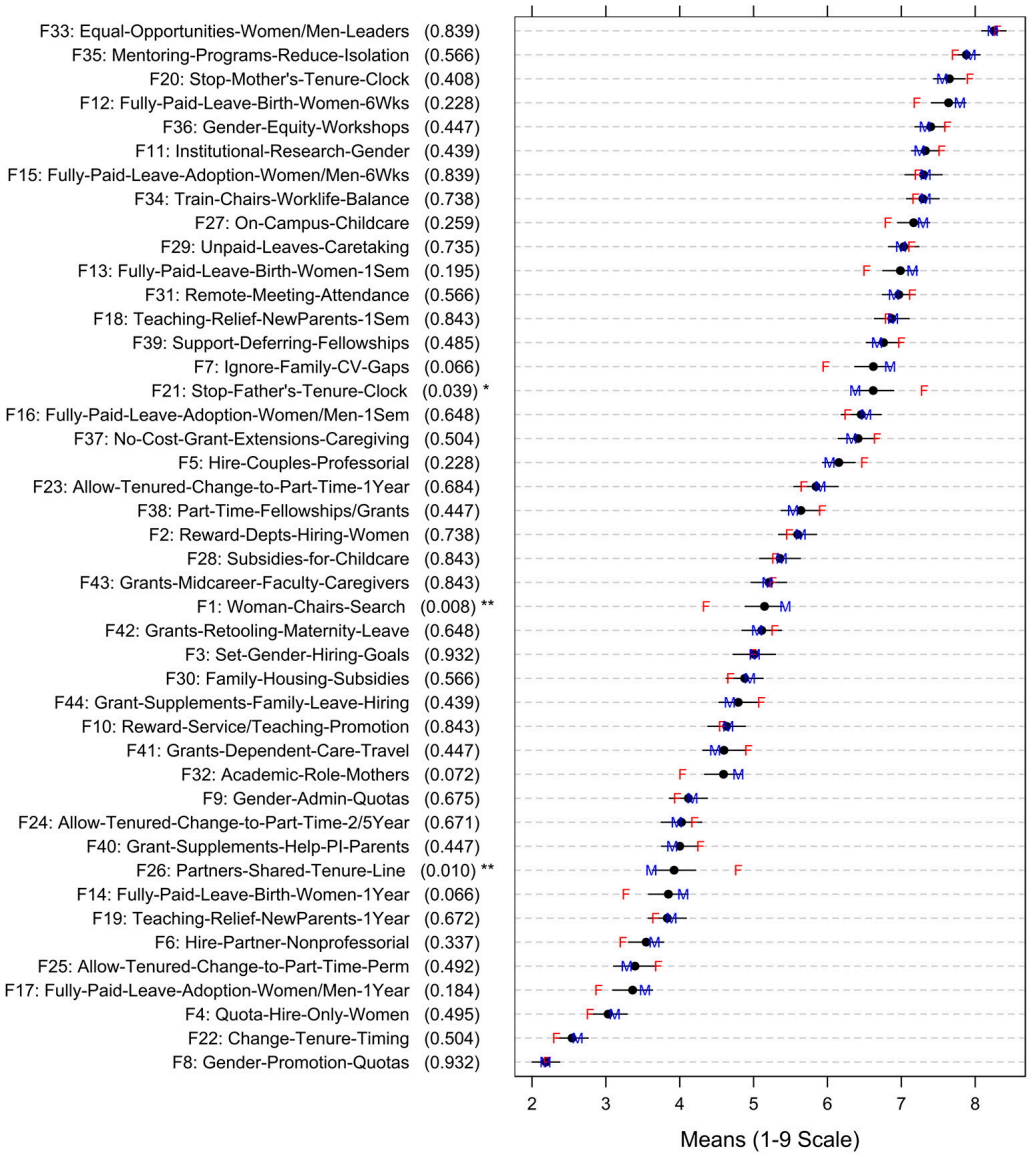
Strategy Feasibility Ratings: Overall Means, Confidence Intervals, and Means by Gender (F = Female; M = Male; adjusted *p*-values in parentheses show significance level of comparison of item ratings by gender; ^*^p ≤ 0.05, ^**^p ≤ 0.01).

The most striking finding in this research was that, overall, women administrators were significantly more likely than men to rate all strategies higher in quality, on average [female mean = 6.33, male mean = 5.99, *t*_(332)_ = −2.58, *p* = 0.01]. This finding suggests that women see the issues of attracting and retaining women in the STEM professoriate as more salient or important than men do, on average. (Figure 3, which shows men's vs. women's mean quality ratings, reveals that women's mean ratings were generally higher than men's.) The feasibility ratings (see Figure 4) did not show this same trend—women *did not* rate the average feasibility of the strategies higher than men did [female mean = 5.51, male mean = 5.55, *t*_(332)_ = 0.34, *p* = 0.74].

To further explore the specific strategies most associated with higher ratings by women, the 44 strategies were analyzed individually to examine gender differences in ratings. Given that in this exploratory process we performed a large number of significance tests, we corrected the Type I error rate. Here, we use the Benjamini-Hochberg False Discovery Rate (FDR) correction, and set the FDR to 5%. In addition, we corrected all *t*-tests for potential violation of homogeneity of variance, and applied the Welch-adjustment to the degrees of freedom, thus making the *p-*values even more conservative. In what follows, we report actual *t-*values and degrees of freedom after Welch's adjustment for significance tests, but only report *p*-values *after* the FDR adjustment. After these conservative adjustments, gender differences in ratings of nine items remained significant. Six of these significant gender effects reflected gender differences in ratings of strategy quality, and three reflected gender differences in ratings of strategy feasibility. We first discuss gender differences in quality ratings, turning next to a consideration of gender differences in feasibility ratings.

### Gender differences in ratings of strategy quality

From the strategy category “Addressing Gender Biases After Hiring,” we found that women more than men supported conducting (and disseminating) institutional research on gender equity [female mean = 7.27, male mean = 6.47, *t*_(197.18)_ = 3.76, *p* = 0.010]. Women's greater emphasis on the importance of gender-equity research is understandable, inasmuch as universities and colleges that conduct and then disseminate such information create an atmosphere in which women's issues are valued, studied, and (hopefully) meaningfully addressed. Obviously, knowing what the issues are is the critical first step in this process.

One cluster of gender differences concerned the role of federal-grant funding—specifically, federal policies, rules, and regulations as potential ways to address issues faced by researchers balancing family and work lives. Under the survey category, “Supporting Greater Flexibility for Federal Grants and Funding,” women rated as higher quality than did men the importance of endorsing supplemental funding for hiring postdocs to maintain momentum during family leaves [female mean = 6.84, male mean = 5.91, *t*_(169.26)_ = 3.39, *p* = 0.011]. Women also were more likely to rate as high quality the strategy of supporting conference and meeting grant supplements to cover cost of PI's dependent care travel (with children's and childcare workers' expenses allowable); female mean = 6.51, male mean = 5.52, *t*_(154.4)_ = 3.27, *p* = 0.011. In a similar vein, women also rated higher than did men the strategy of endorsing federal-grant supplements to offset Principal Investigators' productivity losses due to family-related absences [female mean = 5.76, male mean = 4.66, *t*_(129.31)_ = 3.27, *p* = 0.011]. This group of federal-grant-related strategies reflects new and emerging thinking about how to redefine historical rules that impose highly-limiting restrictions, particularly upon women with children and caretaking responsibilities.

Another widely-cited strategy in the literature for accommodating families with childrearing needs is for universities to support requests for shared tenure lines. From the category, “Attaining Tenure and Maintaining Productivity,” women rated as higher quality than did men the strategy of institutions supporting requests for shared tenure lines (between partners)—female mean = 5.49, male mean = 4.28, *t*_(150.18)_ = 3.58, *p* = 0.01. This finding may reflect greater endorsement by women of the need to truly *balance* family and work life, with implicit compromises affecting both the work and family portions of the balance.

We turn next to a key issue concerning women's retention in the professorial pipeline: Earning tenure, and more specifically, delineating the contents of a tenurable portfolio of work. Obviously, the precise nature of the types of work that are valued during tenure consideration is a critical aspect of the tenure decision, itself. The notion of expanding traditional definitions of what constitutes a tenurable portfolio—to accommodate and value women's styles of working within collaborative settings—also showed a sex difference in level of endorsement in our study. From the category, “Addressing Gender Biases After Hiring,” women administrators were more likely to support the concept of increasing the value of teaching and service plus administration when evaluating a candidate for promotion [female mean = 5.08, male mean = 4.06, *t*_(140.71)_, *p* = 0.011]. This gender difference reflected female administrators' greater desire (as compared to male administrators) to increase the value, during tenure and promotion evaluations, of those tasks undertaken and sometimes prioritized by women faculty.

### Gender differences in ratings of strategy feasibility

Turning to a consideration of the *feasibility of strategies* (as opposed to simply their quality), three strategies emerged as being seen as more feasible by one gender than the other. From the “Attaining Tenure and Maintaining Productivity” category, female administrators saw it as more feasible than did male administrators for *male faculty* to stop the tenure clock for raising children for up to 1 year [female mean = 7.31, male mean = 6.38, *t*_(155.92)_ = 3.05, *p* = 0.039]. Once again, this gender difference reveals that women and men perceive differently men's ability and willingness to delay career advancement in order to prioritize the needs of young children and partners or spouses.

From the category “Addressing Gender Biases During Hiring,” women saw as less feasible the strategy of having a woman chair search committees whenever possible, while men saw this strategy as more feasible [female mean = 4.36, male mean = 5.43, *t*_(171.03)_ = 3.83, *p* = 0.008]. Female administrators' lower-feasibility ratings probably represented an acknowledgment of the sometimes-onerous service and administrative demands placed upon women faculty, particularly in departments in which women are underrepresented.

Echoing an earlier finding, from the category, “Attaining Tenure and Maintaining Productivity,” women administrators saw it as more feasible to support requests for shared tenure lines (between partners) than did male administrators [female mean = 4.79, male mean = 3.62, *t*_(145.91)_ = 3.59, *p* = 0.010]. Note that above we reported that female administrators also saw shared tenure lines as being a higher-quality strategy than did male administrators. Thus, repeatedly we found that female and male administrators held differing views on both the quality and the feasibility of partners sharing work and family duties: Administrators' gender predicted how they rated both the effectiveness of the tenure-line-sharing approach, and the potential for actually accomplishing this approach in the real world.

## General discussion

Women administrators in our sample believed that the 44 strategies for attracting and retaining women faculty were significantly higher in quality overall than was perceived by male administrators. Thus, our findings provide empirical support for the importance of women in administrative roles, since real-world resources are limited and women administrators deem women's recruitment and retention strategies to be generally high in quality and thus more worthwhile than men deem them to be. As can be seen in Figures 3 and 4, there was broad general agreement regarding the *relative quality and relative feasibility of strategies*, with administrators of both genders agreeing in general on the ranking of strategies by quality, or by feasibility. However, women believed the strategies were *higher in quality overall* than men did, although women did not see the strategies as being more *feasible* overall than men did. (In other words, women did not simply use the scale differently from men–women were not more positive in general in all of their ratings, compared with men).

At the level of individual strategies, women and men administrators rated the quality of certain strategies differently, with women rating the following policies as significantly higher in quality than men did: (a) various forms of flexibility with federal-grant funding designed to accommodate women with young children and keep these women in the game; (b) increasing the value of teaching, service, and administrative experience in the tenure/promotion evaluation process; (c) devoting university resources to conducting and disseminating gender-equity research; and (d) supporting requests from partners for shared tenure lines that enable couples to better balance work and personal/caretaking roles. Regarding feasibility of strategies, women administrators saw it as more feasible than men did for men to stop the tenure clock for 1 year due to childrearing demands, and for universities to support requests for shared tenure lines (between partners). But women administrators saw it as less feasible for women to chair search committees, presumably an acknowledgment of the potentially-onerous nature of service demands placed upon women.

What do these findings mean for the debate about how to attract and retain more women in academic science? Our national survey revealed that women administrators think differently from their male counterparts about certain key approaches to attracting and retaining women. Because women administrators value pro-women strategies more than men do overall, and value some individual strategies in particular more than men do, the resources lobbied for and allocated by these women administrators may be deployed more often toward the strategies they endorse, although we offer no specific evidence confirming this. Endorsement of a strategy in a survey may not necessarily translate into action. Likewise, the opinion of an administrator does not necessarily mean that the effectiveness (should the actual strategy be implemented in policy) is proven; the present data consisted of ratings by administrators. The current survey was not designed to address whether an administrator actually implemented these strategies or how successful they were.

Women administrators' views that the strategies are higher in quality overall than men perceive them to be could result in women administrators spending relatively more of their limited budgets than men would on women-in-science issues. It has been argued that women and people of color in academic administrative posts bring different perspectives to their jobs (Smith et al., 2004), and our data support this position, at least with regard to beliefs about the quality and feasibility of strategies for attracting and retaining women in the STEM professoriate.

It is worth noting, however, that men and women agreed much of the time about the relative ranking of strategies—in other words, both genders basically agreed on what constituted the best vs. worst strategies among the 44 presented for evaluation. Women in general endorsed the strategies as being higher in quality overall than did men, and women and men disagreed about the quality of some strategies, but there was general agreement about the overall quality of one strategy relative to all the others, as seen by the similar rank ordering of strategies by both sexes. Administrators basically agreed on what represented higher- vs. lower-quality strategies. This is heartening news, since agreement about what constitutes a good strategy generally makes it simpler to get the strategy actually introduced as a policy. But women administrators were more supportive of strategies to attract and retain women in STEM, overall—and furthermore, there were some specific strategies that women endorsed at a significantly higher level than their male counterparts did.

Our data suggest that there are a substantial number of highly-rated strategies, and call into question the potential endorsement of the low-rated strategies. The highest-quality strategy was to provide on-campus childcare centers (rated *M* = 8.36 out of 9). Providing equal opportunity for women and men to lead committees and research groups was next (*M* = 8.26), followed by developing mentoring programs to reduce isolation of female faculty (*M* = 7.92) and stopping the tenure clock for raising children for up to 1 year per child (*M* = 7.59). There was broad support for providing fully-paid leave for giving birth for tenure-track women only for one semester (*M* = 7.52) and for allowing unpaid sabbaticals and leave of absences for both genders without penalty, for family-related reasons such as elder caretaking and issues with children (*M* = 7.50). Training department chairs on helping faculty manage work-life issues (*M* = 7.40) was seen as a high-quality strategy, as was supporting the deferred start of fellowships to allow for caregiving (*M* = 7.20), and providing teaching relief for new tenure-track parents for one semester (*M* = 7.17). Additional high-quality strategies involved supporting no-cost extensions for caregiving on grants and fellowships (*M* = 7.12), and exploring and endorsing couples-hiring (*M* = 7.05). Providing fully-paid leave for adoption/new parenthood (for tenure-track women and men), for one semester, was also seen as valuable (*M* = 7.03).

The two lowest-rated strategies involved use of gender quotas for hiring (*M* = 3.62) and promotion (*M* = 2.46). Interestingly, one strategy that has been widely recommended for its potential to alleviate the conflict between women's biological clock and the tenure clock—changing the timing of tenure assessment not to coincide with peak fertility and childrearing demands—was also rated as a relatively poor idea (*M* = 3.83), and this was true for administrators of both genders. Similarly, allowing professors to change from full-time to part-time, permanently, on the tenure track—another strategy often acknowledged as being potentially beneficial to women—was also rated as low in quality (*M* = 3.97). It is fascinating that so many strategies widely written about and discussed as being potentially helpful were nevertheless viewed by active administrators as being low in quality.

Overall, the take-home message of this national empirical study was that (a) female administrators perceive strategies to retain women STEM professors as being higher in quality overall–i.e., more important and worthy—than do male administrators, (b) women vs. men administrators perceive some strategies differently; i.e., women and men disagree about the quality of certain strategies, and (c) women and men administrators agree in general regarding which strategies are higher vs. lower in quality. Thus, the belief that women in administrative roles will place greater emphasis than men will on strategies to retain women STEM professors was supported. A hopeful result was that men and women agree in general about better vs. worse approaches—thus suggesting that committees comprised of people of both genders will be able to find common ground for selecting and funding potential strategies. However, there were important exceptions; for example, women's greater endorsement of the need to more heavily weigh teaching, service, and administration in tenure-decision-making, and women's greater support of shared tenure lines (between partners) to enable broader sharing of childrearing and work activities and goals within a family. Areas of disagreement regarding strategy quality are important focuses of future policy and planning, because female administrators may have insights into how to retain women professors, by virtue of their personal experiences, that male administrators do not share.

## Ethics statements

Cornell University IRB approved this project. Fully informed consent was given.

## Author contributions

WW designed the study, supervised data collection and analysis, interpreted findings, and wrote the manuscript. AM collected data and did preliminary analyses and write-up. SB assisted with survey content/development. BC assisted with follow-up data collection. FT and FV analyzed and interpreted data, and FT created the figures. SC helped design the study, interpret findings, and write the manuscript.

## Funding

This research was supported by a grant from the NIH Grant 1R01NS069792-01.

### Conflict of interest statement

The authors declare that the research was conducted in the absence of any commercial or financial relationships that could be construed as a potential conflict of interest.
